# Supplementary material for: G-quadruplexes promote the motility in MAZ phase-separated condensates to activate CCND1 expression and contribute to hepatocarcinogenesis
Source: Nat Commun. 2024 Feb 5;15:1045. doi: 10.1038/s41467-024-45353-5 (PMC10844655; doi:10.1038/s41467-024-45353-5)
Supplement: Supplementary file 1 — Supplementary Information [file 41467_2024_45353_MOESM1_ESM.pdf]

Supplementary Data

Supplementary Figures

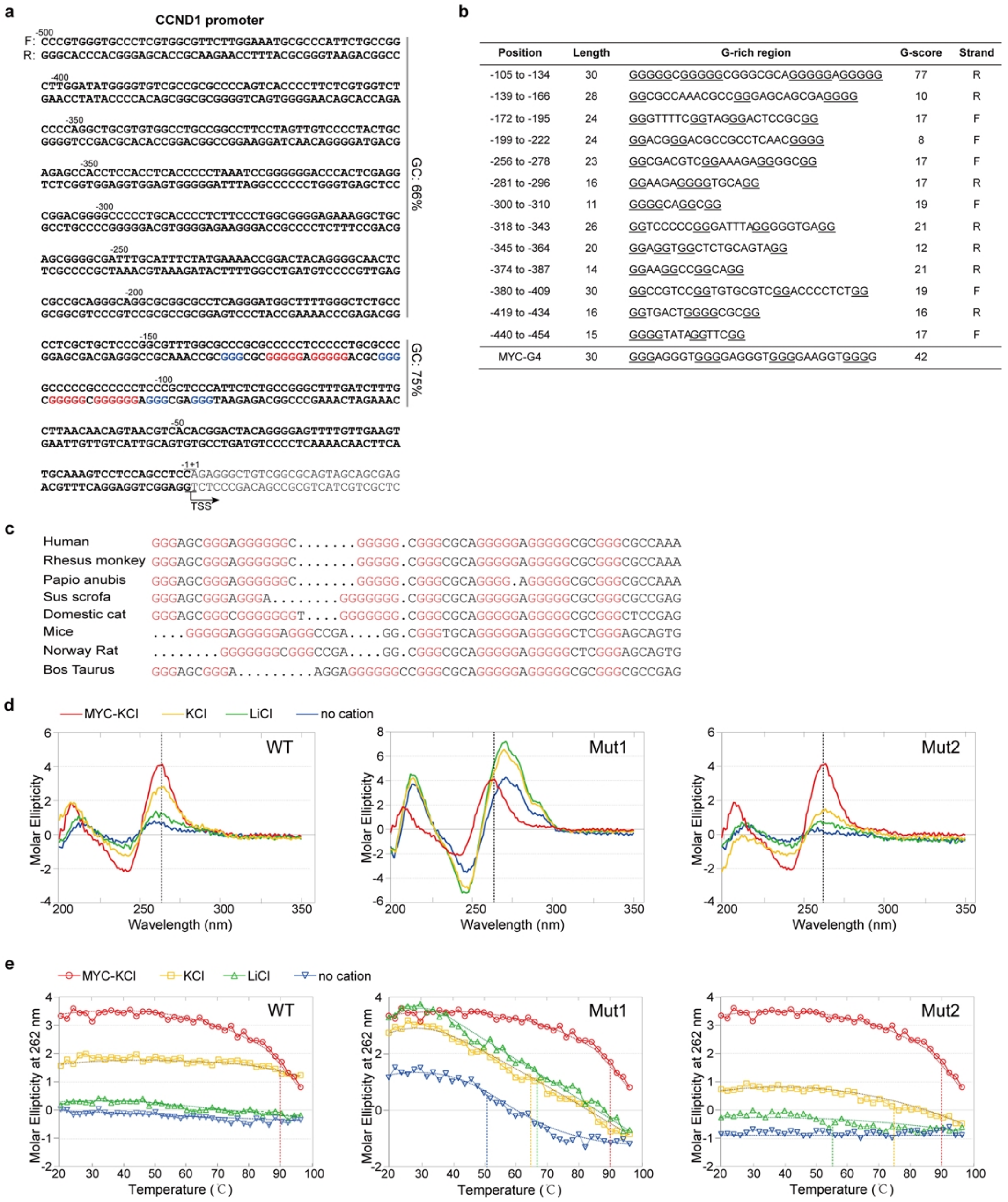

Supplementary Figure 1. Analysis of CCND1 promoter sequences related to G4 formation.

a Sequence from −500 to +28 nt in the CCND1 promoter with the transcription start site (TSS) designated as "+1" and its first upstream nt as "-1". The eight consecutive G-tracts on the negative strand are shown in red (≥5G) and blue (3G) letters.

b The analysis of the -500 to -1 nts region of the human CCND1 promoter for the putative G4 motifs using the QGRS Mapper (https://bioinformatics.ramapo.edu/QGRS). G-tracts involved in G4

formation are underlined.

c Alignment of the G-rich regions in the negative strands of the CCND1 promoters in different vertebrate species. The region corresponds to the -142 to -92 nts in the human CCND1 promoter. The G-tracts are shown in red letters.

d CD spectrometric analyses of oligos as labeled and annealed under the conditions without extra cation, or in the presence of 50 mM KCl or LiCl (*n* = 3 biologically independent experiments). The dashed lines denote the signature molar ellipticity peaks of parallel G4s at 262 nm.

e CD spectrometric analysis of thermal melting stability at 262 nm for the annealed oligos in the absence or presence of KCl or LiCl (*n* = 3 biologically independent experiments). The dashed lines indicated the theoretical Tm values.

Source data are provided as a Source Data file.

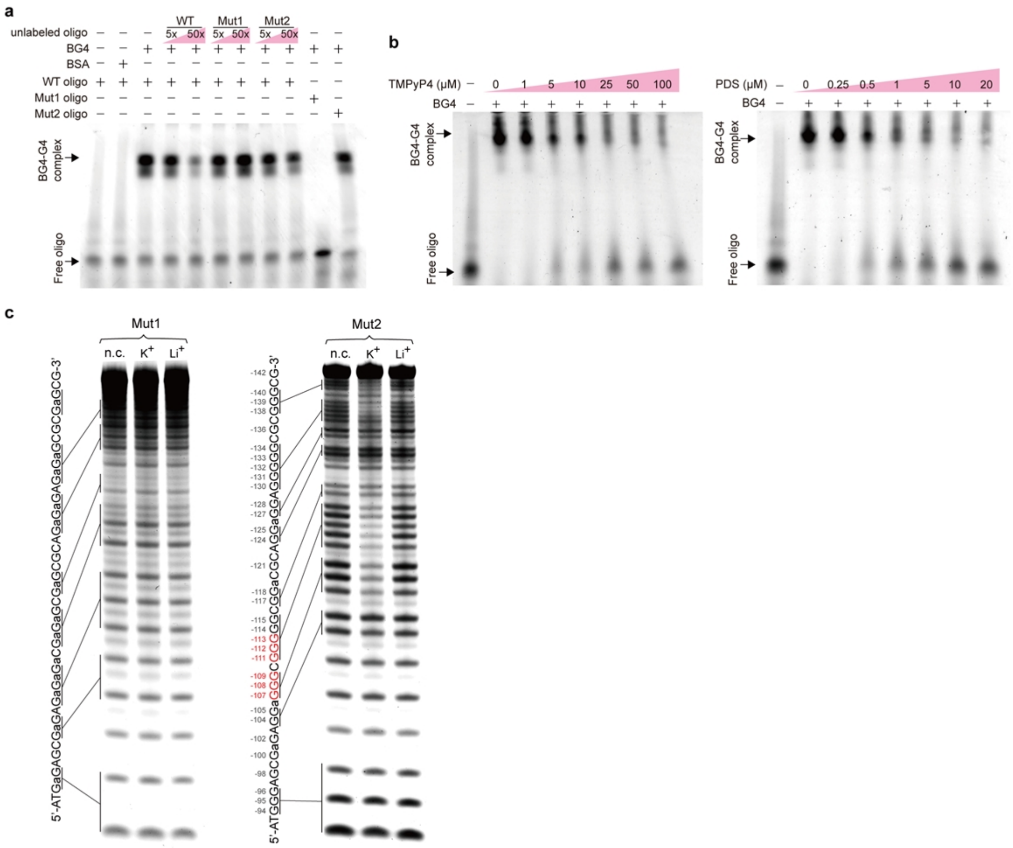

Supplementary Figure 2. Additional data to verify G4 formation in the CCND1 promoter.

a EMSA analysis of BG4 binding to the FAM-labeled oligos. In EMSA studies, 200 nM of each oligo annealed in the presence of KCl and 300 ng of BG4 were used. In competitive binding reactions, 5× and 50× of annealed unlabeled oligos were added to compete with cognate labeled oligos for the binding. The positions of BG4-G4 complex and free oligos are denoted.

b EMSA analysis of PDS and TMPyP4 effects on BG4 binding to the FAM-labeled WT oligos. In the assays, 200 nM oligos and 300 ng of BG4 were used. Oligos were annealed in the presence of KCl, and increasing amounts of TMPyP4 or PDS were added into the annealed oligos prior to BG4 addition. The positions of BG4-G4 complex and free oligos are denoted.

c DMS footprinting assays of CCND1-G4 mutants (Mut1 and Mut2). FAM-labelled Mut1 and Mut2 oligos were annealed in the absence or presence of KCl or LiCl, and treated by the procedure described in the Methods section. The corresponding DNA sequences were labeled with their positions on the CCND1 promoter, and the protected guanines are shown in red letters.

In (**a-c**), data are representative of three biologically independent experiments.

Source data are provided as a Source Data file.

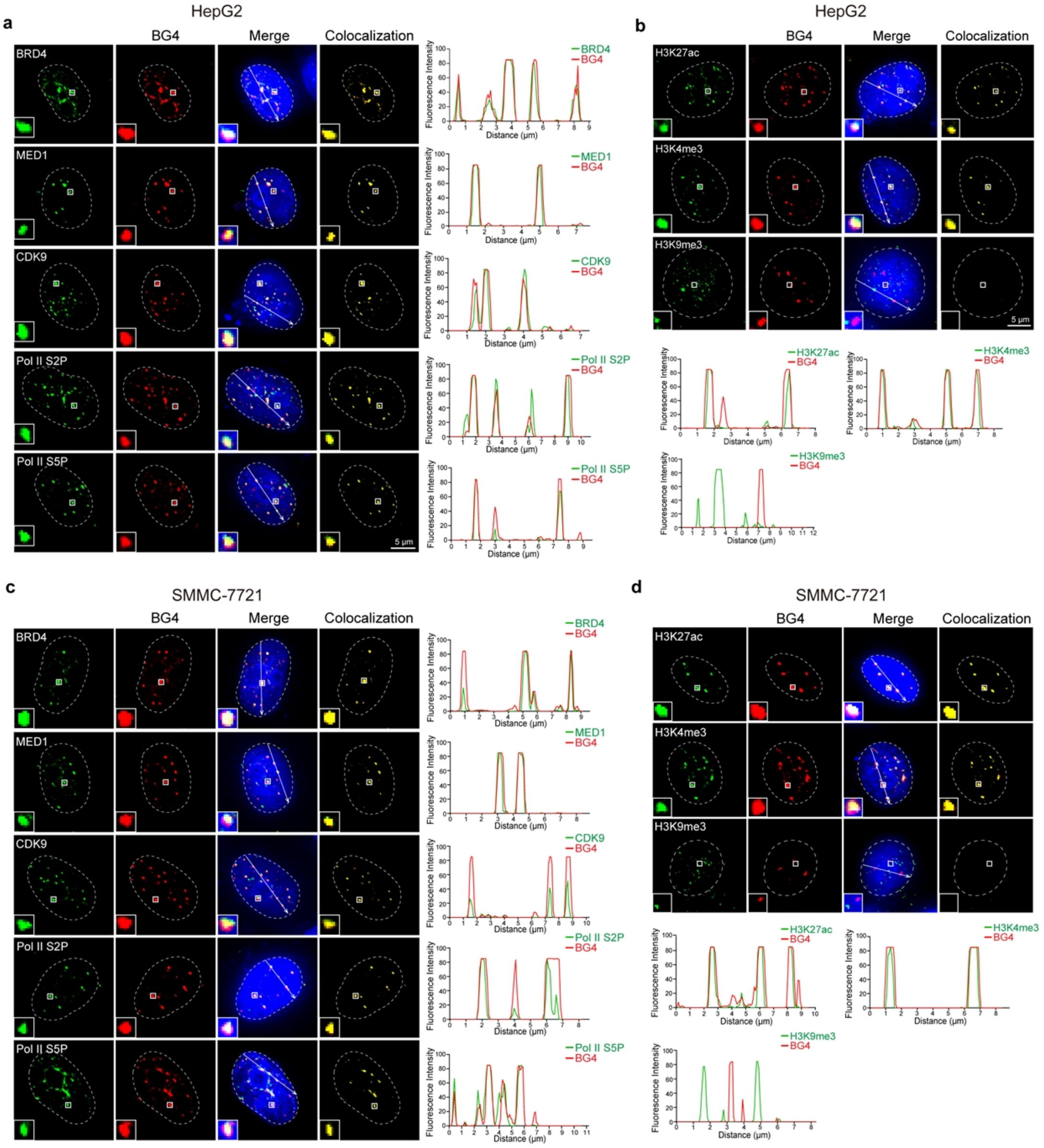

Supplementary Figure 3. Colocalization studies of BG4 with transcriptional coregulators.

HepG2 (**a**, **b**) and SMMC-7721 (**c**, **d**) cells were stained with BG4 and individually co-stained with indicated antibodies. Representative of three biologically independent experiments.

Colocalization of BG4 (red) and BRD4, MED1, CDK9, active RNA Pol II S2P/S5P, H3K27ac, H3K4me3 and H3K9me3 (green) in HepG2 and SMMC-7721 cells was evaluated. Line scans of colocalization images are depicted by white profile arrow lines with quantification shown at right (**a**, **c**) or bottom (**b**, **d**).

Source data are provided as a Source Data file.

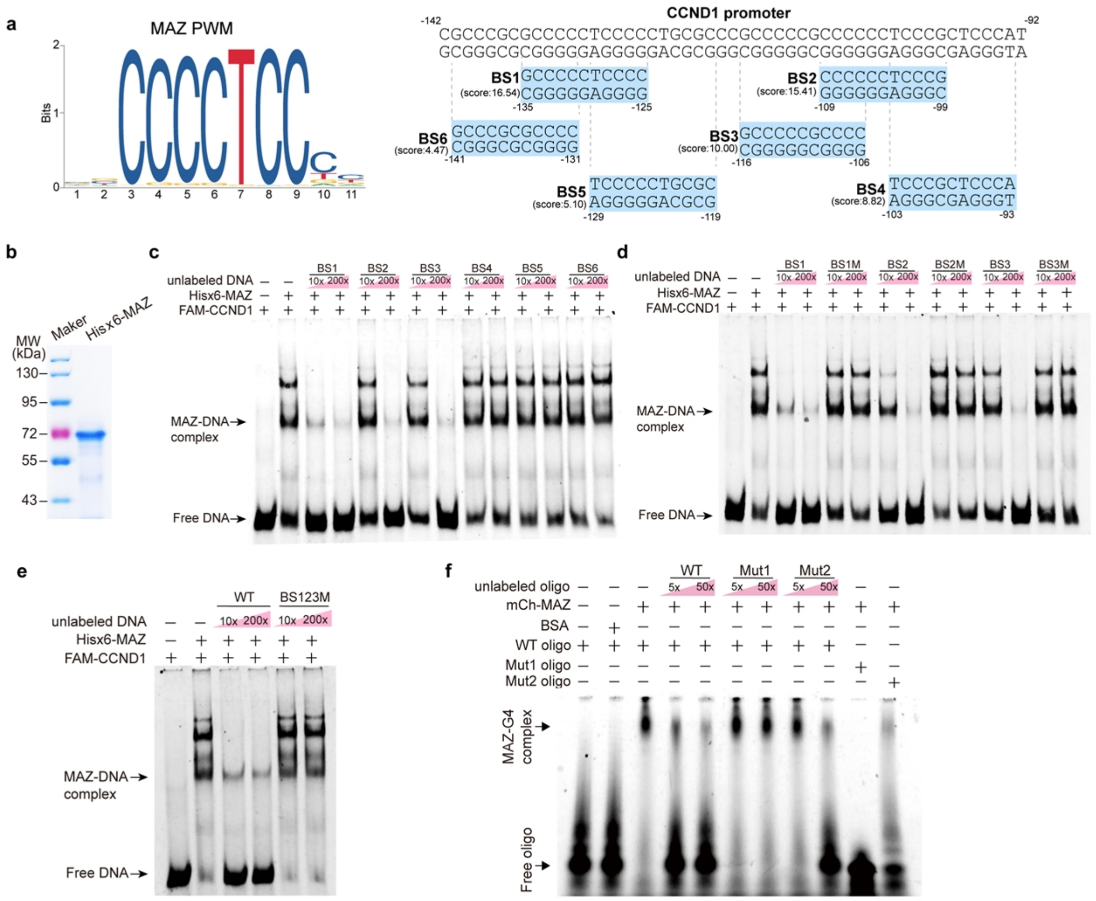

Supplementary Figure 4. Analysis of MAZ binding sites in the G4-forming sequences of the CCND1 promoter, and verification of MAZ binding to the FAM-labeled oligos.

a The conserved DNA-binding elements of the MAZ protein with a core sequence of CCCCTCC based on the Jaspar Matrix database (ID: MA1522.1) (Left), and the putative MAZ binding sites in the G4 forming region (-142 to -92 nts) of the negative strand in the CCND1 promoter (Right).

b SDS-PAGE analysis of purified recombinant His×6-MAZ.

c-e EMSA analyses of unlabeled DNA in competing with labeled DNA of each putative MAZ binding sites in the CCND1 promoter in binding to purified His×6-MAZ. Excessive unlabeled doubled-stranded DNA BS1-BS6 (by annealing two complementary oligos) containing original sequences (**c**), individually (**d**) or BS1/2/3 combinatorially (**e**) mutated MAZ binding elements of the CCND1 promoter were mixed with FAM-labeled corresponding DNA as indicated and purified His×6-MAZ. The sequences of BS1-BS6, BS1M, BS2M, BS3M and BS123M are shown in Supplementary Table 2.

f EMSA analysis of MAZ binding to the FAM-labeled oligos. In EMSA studies, 200 nM of each

oligo annealed in the presence of KCl and 300 ng of mCherry-MAZ were used. In competitive binding reactions, 5× and 50× of annealed unlabeled oligos were added to compete with cognate labeled oligos for the binding. The positions of MAZ-G4 complex and free oligos are denoted.

In (**b-f**), data are representative of three biologically independent experiments.

Source data are provided as a Source Data file.

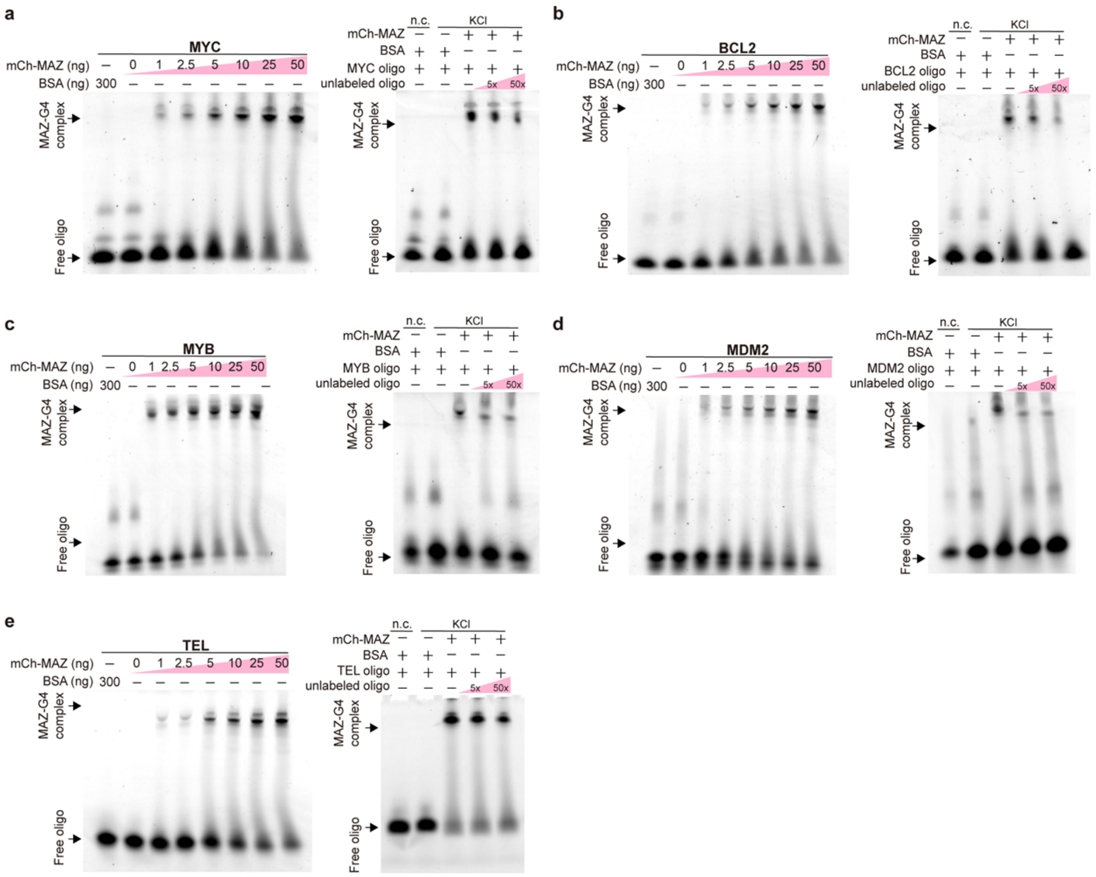

Supplementary Figure 5. EMSA analysis of MAZ binding to well-defined G4s in different oncogenes. FAM-labeled oligos of the G4 motifs in the MYC (**a**), BCL2 (**b**), MYB (**c**), MDM2 (**d**) promoters and telomere (TEL) (**e**), were mixed with increasing amounts of purified recombinant MAZ, followed by analyses of native polyacrylamide gel electrophoresis (left panel of each). The competition of unlabeled oligos with the FAM-labeled oligos to MAZ was also tested (right panel of each). Data are representative of three biologically independent experiments. The oligo sequences derived from these oncogenes or telomere are shown in Supplementary Table 2.

Source data are provided as a Source Data file.

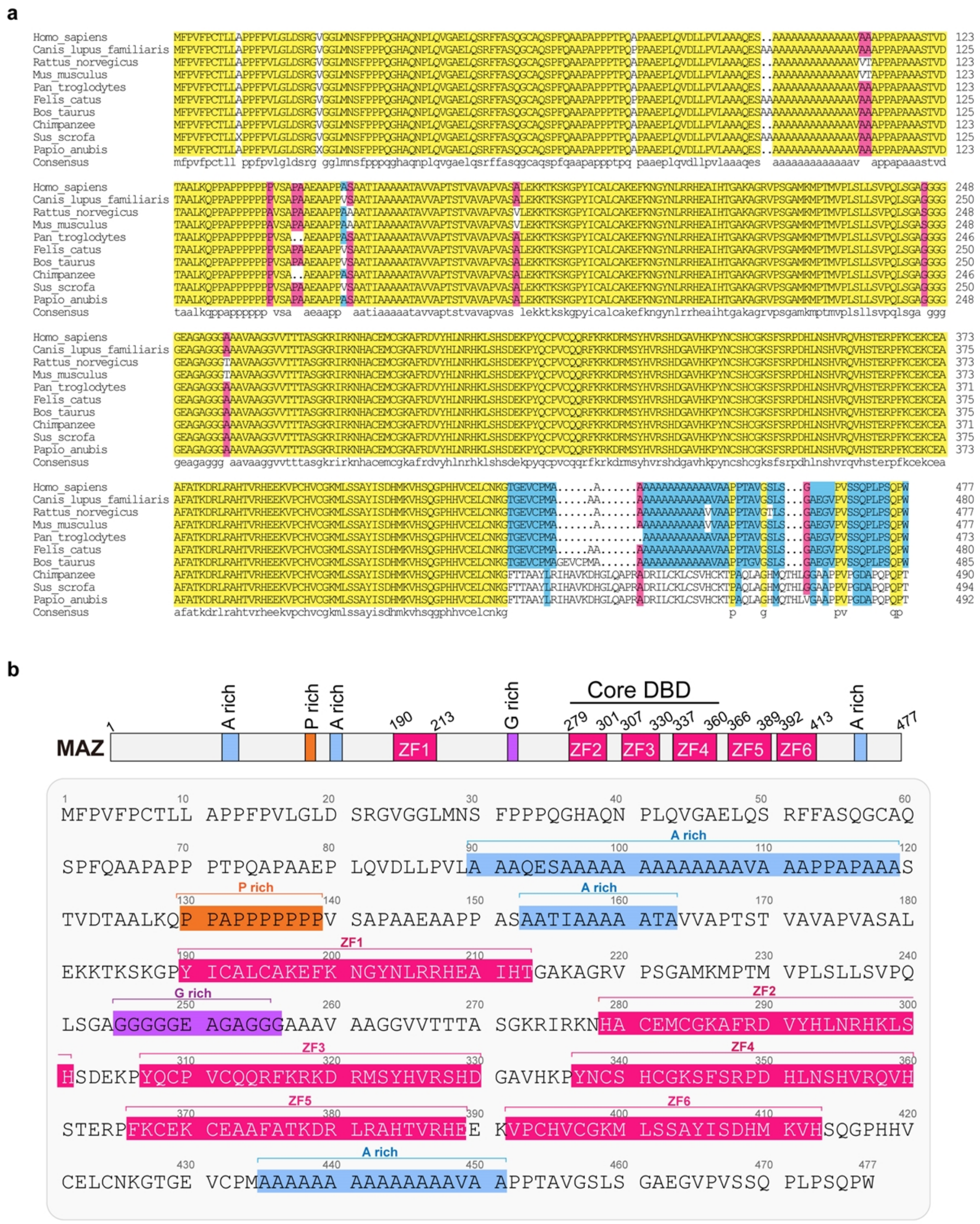

Supplementary Figure 6. Alignment MAZ proteins from different vertebrate species (a), the domain structures and amino acid sequence of human MAZ protein (b). In (**a**), homologous regions among these MAZ proteins are highlighted with consensus sequences at the bottom line. In (**b**), three alanine (A) rich regions are highlighted in blue color, proline (P) rich region is in orange, glycine (G) rich region is in purple, and six zinger fingers (ZFs) is in pink. Core DBD: core DNA binding domain.

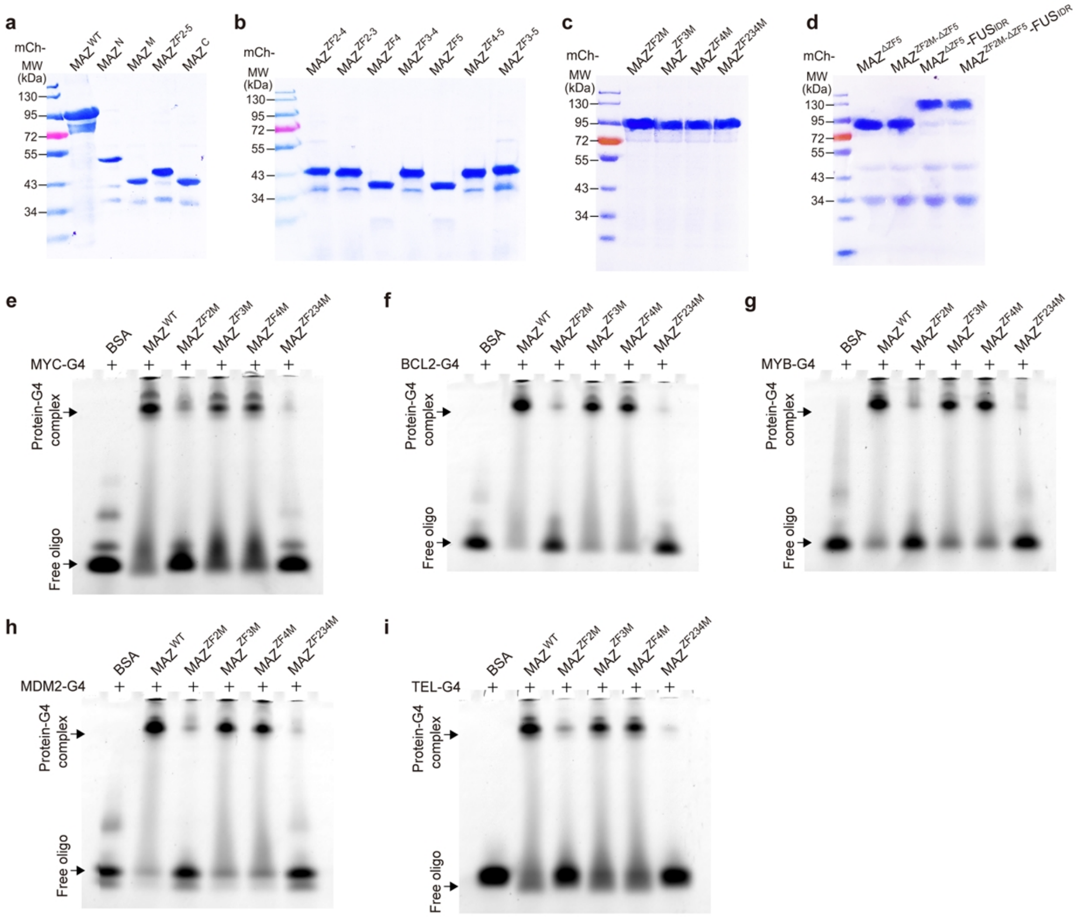

Supplementary Figure 7. SDS-PAGE analyses of purified recombinant MAZ proteins and EMSA studies of MAZ proteins' binding to well-defined G4s.

a-d SDS-PAGE analyses of purified recombinant MAZ WT and its mutant proteins used in this study.

e-i EMSA analyses of recombinant MAZ-WT, ZF2M, ZF3M, ZF4M and ZF234M binding to FAM-labeled G4 oligos derived from the MYC (**e**), BCL2 (**f**), MYB (**g**), MDM2 (**h**) promoters, and telomere (TEL) (**i**). BSA was used as a control. The sequences of the G4 oligos are shown in Supplementary Table 2.

In (**a-i**), data are representative of three biologically independent experiments.

Source data are provided as a Source Data file.

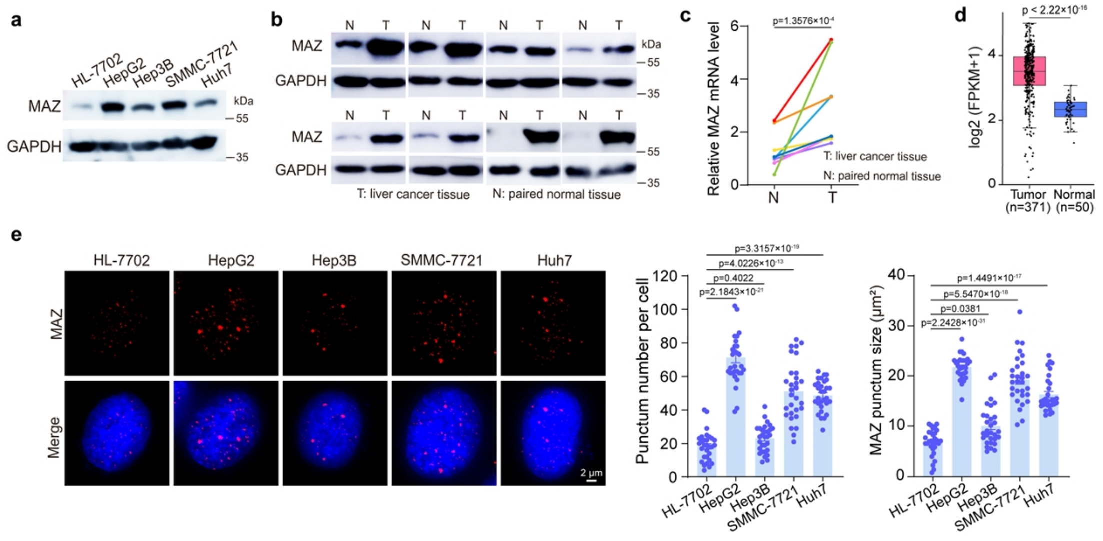

Supplementary Figure 8. MAZ expression in liver cancer and its immunofluorescence staining of MAZ in HL-7702 cells and four HCC cells.

a-d MAZ expression in liver cells and cancer tissues. In (**a**, **b**), Western blot analyses to compare MAZ protein expression between normal liver HL-7702 cells and four HCC cell lines (**a**), and between 8 normal liver cancer samples and correspondingly matched para-cancerous normal liver tissues (**b**). In (**c**, **d**), MAZ expression from RT-qPCR analyses of the 8 pairs of liver cancer and the matched para-cancerous normal tissues (**c**), and the analysis of a TCGA Liver Hepatocellular Carcinoma (TCGA-LIHC) dataset consisting of 371 cancer samples and 50 para-carcinoma tissues (**d**). In (**a-c**), data are representative of three biologically independent experiments.

e Immunofluorescence staining in HL-7702 cells and four HCC cells using the MAZ antibody. The quantification of nuclear puncta for their numbers and sizes is shown at right. Data are mean ± s.e.m. (n = 30 cells each). Unpaired two-tailed Student's t-test was used for statistical analysis.

Source data are provided as a Source Data file.

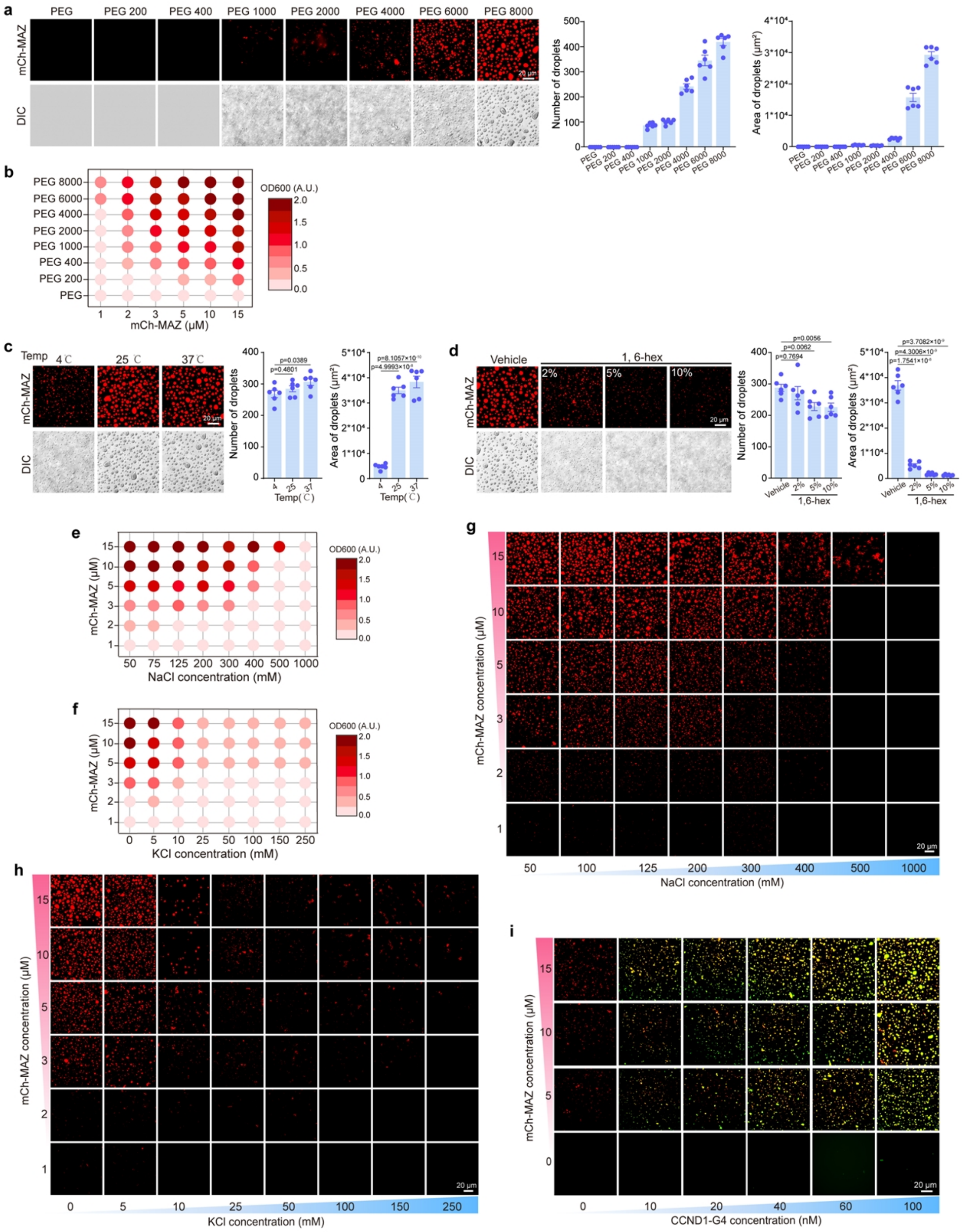

Supplementary Figure 9. Characterization of MAZ droplet formation *in vitro*.

a-d *In vitro* droplet formation assays of mCherry-MAZ protein in PEGs with different molecular weights (**a**, **b**), at different temperatures (**c**), and in buffers containing different amounts of 1,6-hexanediol (1,6-hex) (**d**).

In **a**, **c**, **d**, the images at left are the representative fluorescence and DIC images of the droplets. The bar graphs at right are the quantification of droplets' numbers and area. Data are mean ± s.e.m. (n =

6 fields each). In (**c**, **d**), unpaired two-tailed Student's *t*-test was used for statistical analysis.

e-h Assays to determine MAZ droplet formation in buffers containing different concentrations of NaCl and KCl. In (**e**, **f**), phase diagrams of turbidity for mCherry-MAZ at different concentrations in buffers containing different concentrations of NaCl (**e**) and KCl (**f**). In (**g**, **h**), representative fluorescence droplet images of (**e**) and (**f**), respectively, are presented.

i Fluorescence droplet images of mCherry-MAZ in different concentrations were mixed with FAM-labeled CCND1-G4 oligo (WT).

Scale bar, 20 μm (**a**, **c**, **d**, **g-i**).

In (**b**, **e-i**), data are representative of three biologically independent experiments.

Source data are provided as a Source Data file.

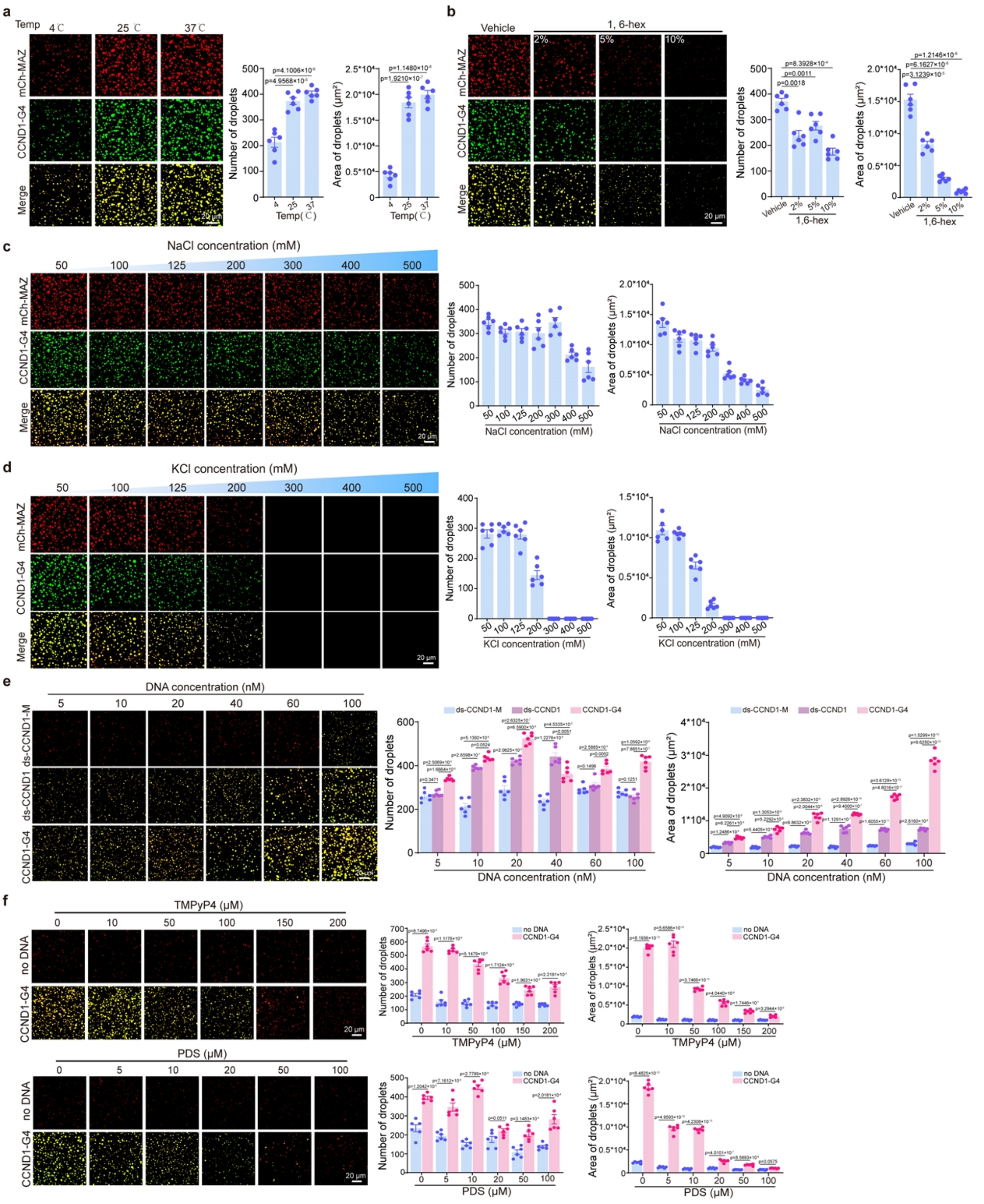

Supplementary Figure 10. Regulation of droplet formation by MAZ and CCND1-G4 *in vitro*.

a-d Droplet formation assays of mCherry-MAZ protein in the presence of the CCND1-G4 WT oligo at different temperatures (**a**), in buffers with different concentrations of 1,6-hex (**b**), in buffers with different concentrations of NaCl (**c**), or KCl (**d**).

e Effects of the double-stranded (ds) oligo duplex derived from the MAZ binding sites in the CCND1 promoter (ds-CCND1), its mutated sequence (ds-CCND1-M), and annealed CCND1-G4 WT oligos on the mCherry-MAZ's droplet formation. The sequence of ds-CCND1 and ds-CCND1-M are provided in Supplementary Table 2.

f Effects of TMPyP4 and PDS on mCherry-MAZ/CCND1-G4 *in vitro* droplet formation.

In **a-f**, representative fluorescence images of the droplets are shown at left, while quantification of droplets' numbers and area are shown at right. Data are mean ± s.e.m. (*n* = 6 fields each).

In (**a**, **b**, **e**, **f**), unpaired two-tailed Student's *t*-test was used for statistical analysis. Scale bar, 20 μm. Source data are provided as a Source Data file.

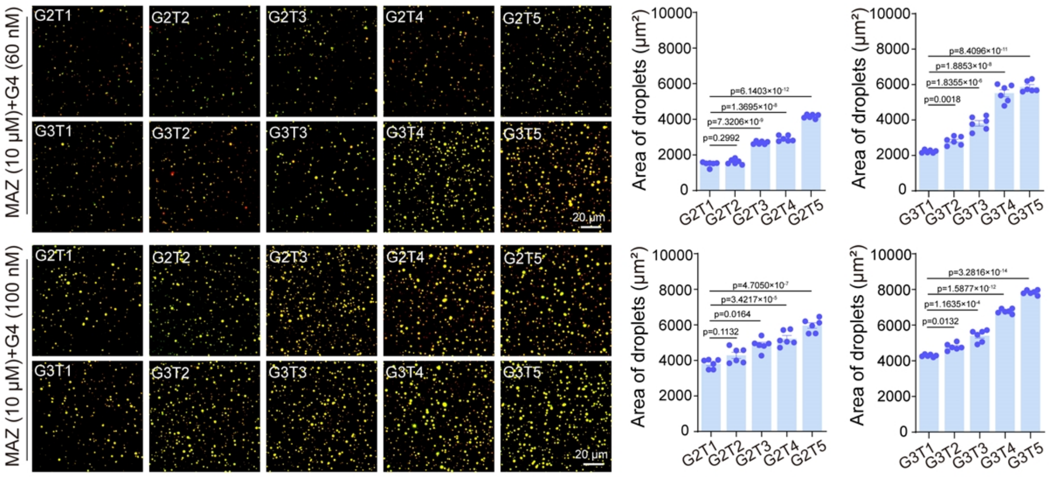

Supplementary Figure 11. Effects of G4s with different G-quartet numbers and loop lengths on MAZ/G4 droplet formation.

The concentrations of mCherry-MAZ and FAM-labelled G4-forming oligos were indicated at left. The sequences tested oligos are shown in Supplementary Table 4. Representative fluorescence images of the droplets are shown at left, while quantification of droplets' area is shown at right. Data are mean ± s.e.m. (*n* = 6 fields each). Unpaired two-tailed Student's *t*-test was used for statistical analysis. Scale bar, 20 μm.

Source data are provided as a Source Data file.

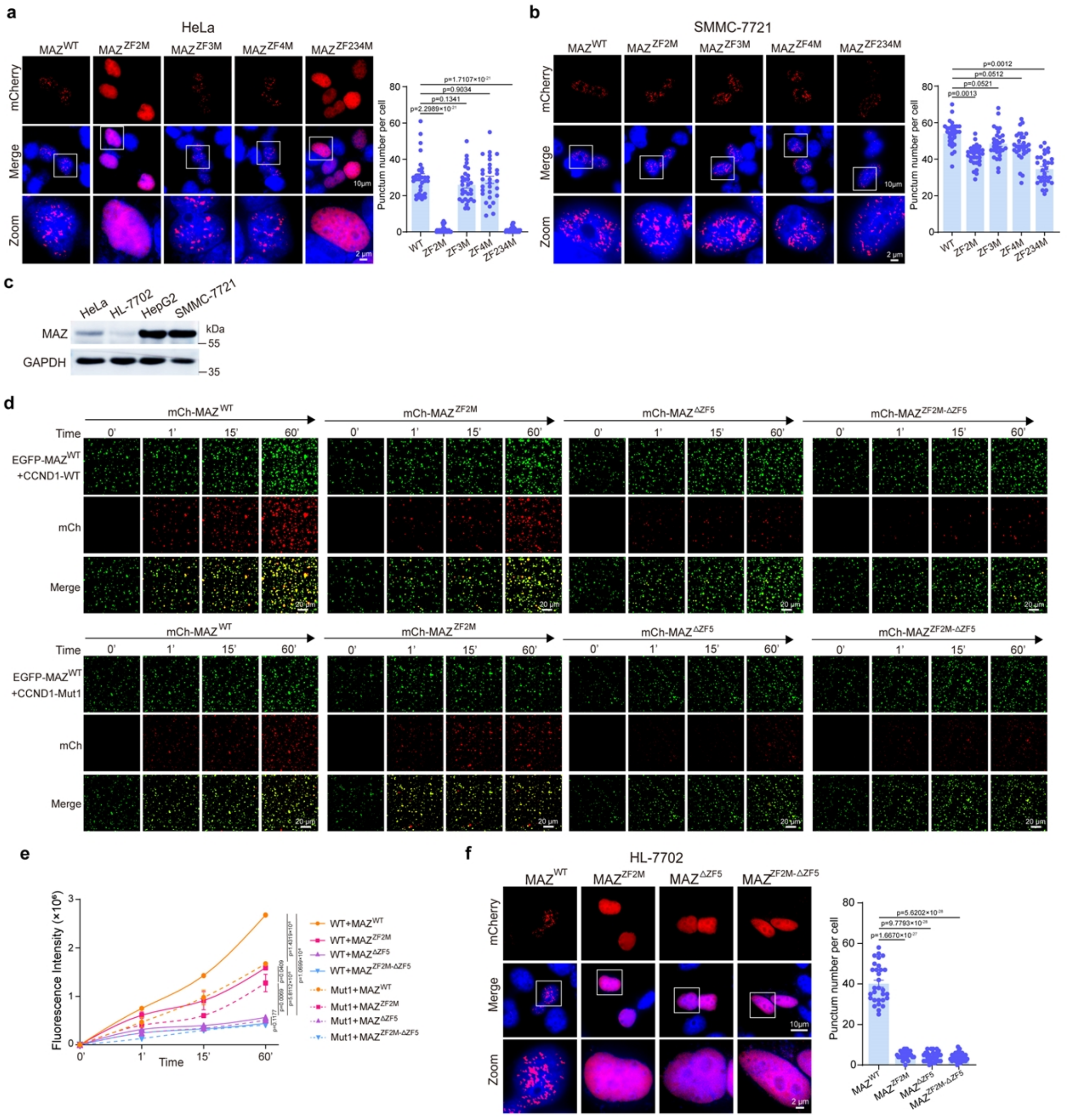

Supplementary Figure 12. Determination of punctum formation by MAZ WT and mutants in cells, and verification of the mechanism of MAZ/G4 condensate formation *in vitro*.

a, **b** Punctum formation of MAZ^{WT}, MAZ^{ZF2M}, MAZ^{ZF3M}, MAZ^{ZF4M} and MAZ^{ZF234M} in HeLa (**a**), and SMMC-7721 (**b**) cells. Representative images of fluorescence signals in cells are shown at left, while the quantification of the puncta is shown at right.

c Western blot analysis of relative endogenous MAZ protein levels in different cell lines. Representative of three biologically independent experiments.

d Time-lapse imaging of integration of mCherry-MAZ WT and mutants (red) into pre-formed droplets by EGFP-MAZ and CCND1-G4 WT (upper panel) or Mut1 (lower panel) (green). Scale bar, 20 μm.

e Fluorescence quantification of the merged images in (**d**). Data are presented as mean ± s.d. (*n* = 3 biologically independent experiments). Unpaired two-tailed Student's *t*-test was used for statistical analysis.

f Punctum formation of MAZ^{WT}, MAZ^{ZF2M}, MAZ^{ZF3M}, MAZ^{ZF4M} and MAZ^{ZF234M} in HL-7702 cells. Representative images of fluorescence signals in cells are shown at left, while the quantification of the puncta is shown at right.

In (**a**, **b**, **f**), data are presented as mean ± s.e.m. (*n* = 30 cells each). Unpaired two-tailed Student's *t*-test was used for statistical analysis.

Source data are provided as a Source Data file.

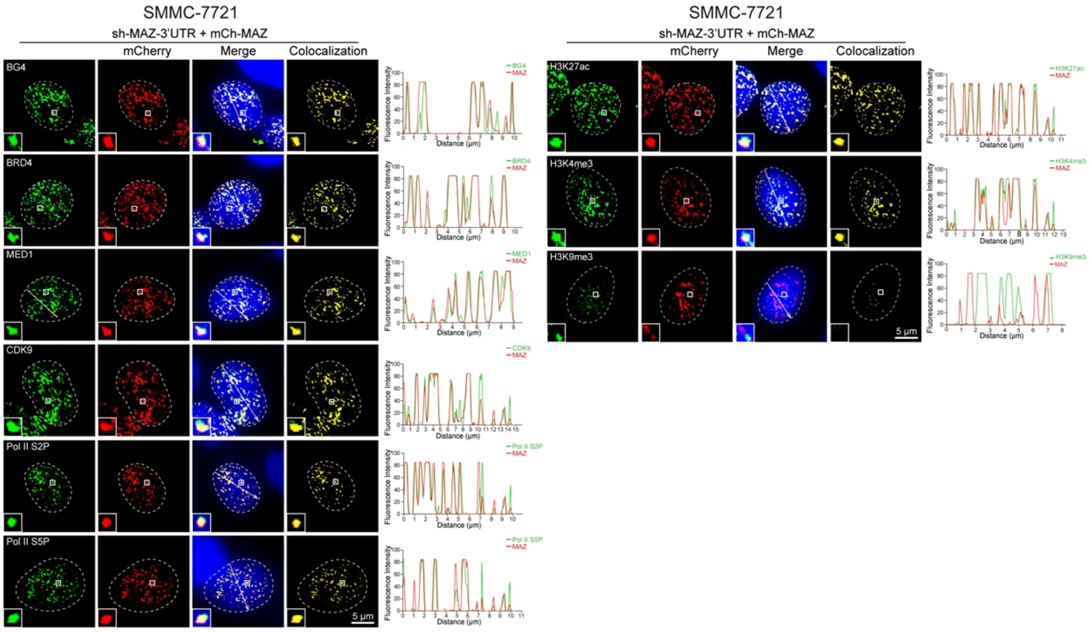

Supplementary Figure 13. Analysis of mCherry-MAZ colocalization with transcriptional coregulators in SMMC-7721 cells.

SMMC-7721 cells were infected by lentivirus carrying sh-MAZ-3'UTR to knock down endogenous MAZ and then transfected by mCherry-MAZ plasmid. With the mCherry-MAZ in red, the cells were stained by antibodies against G4 (BG4), BRD4, MED1, CDK9, active RNA Pol II S2P/S5P, H3K27ac, H3K4me3 and H3K9me3 (in green). Line scans of colocalization images are depicted by white profile arrow lines with quantification shown at right. Representative of three biologically independent experiments.

Source data are provided as a Source Data file.

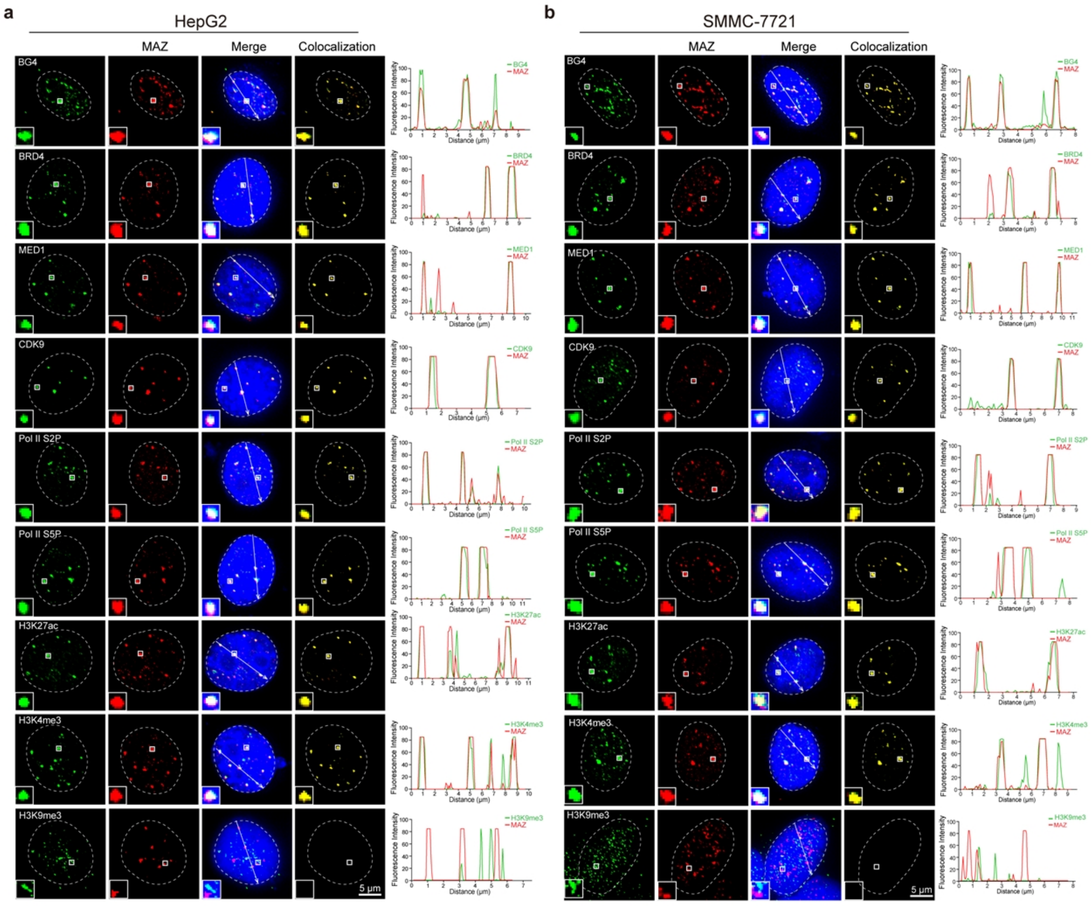

Supplementary Figure 14. Analysis of endogenous MAZ colocalization with transcriptional coregulators in HepG2 and SMMC-7721 cells.

HepG2 (**a**) and SMMC-7721 (**b**) cells were co-stained by the MAZ antibody (red) and one of the antibodies against G4 (BG4), BRD4, MED1, CDK9, active RNA Pol II S2P/S5P, H3K27ac, H3K4me3 and H3K9me3 (green). Line scans of colocalization images are depicted by white profile arrow lines with quantification shown at right. Representative of three biologically independent experiments.

Source data are provided as a Source Data file.

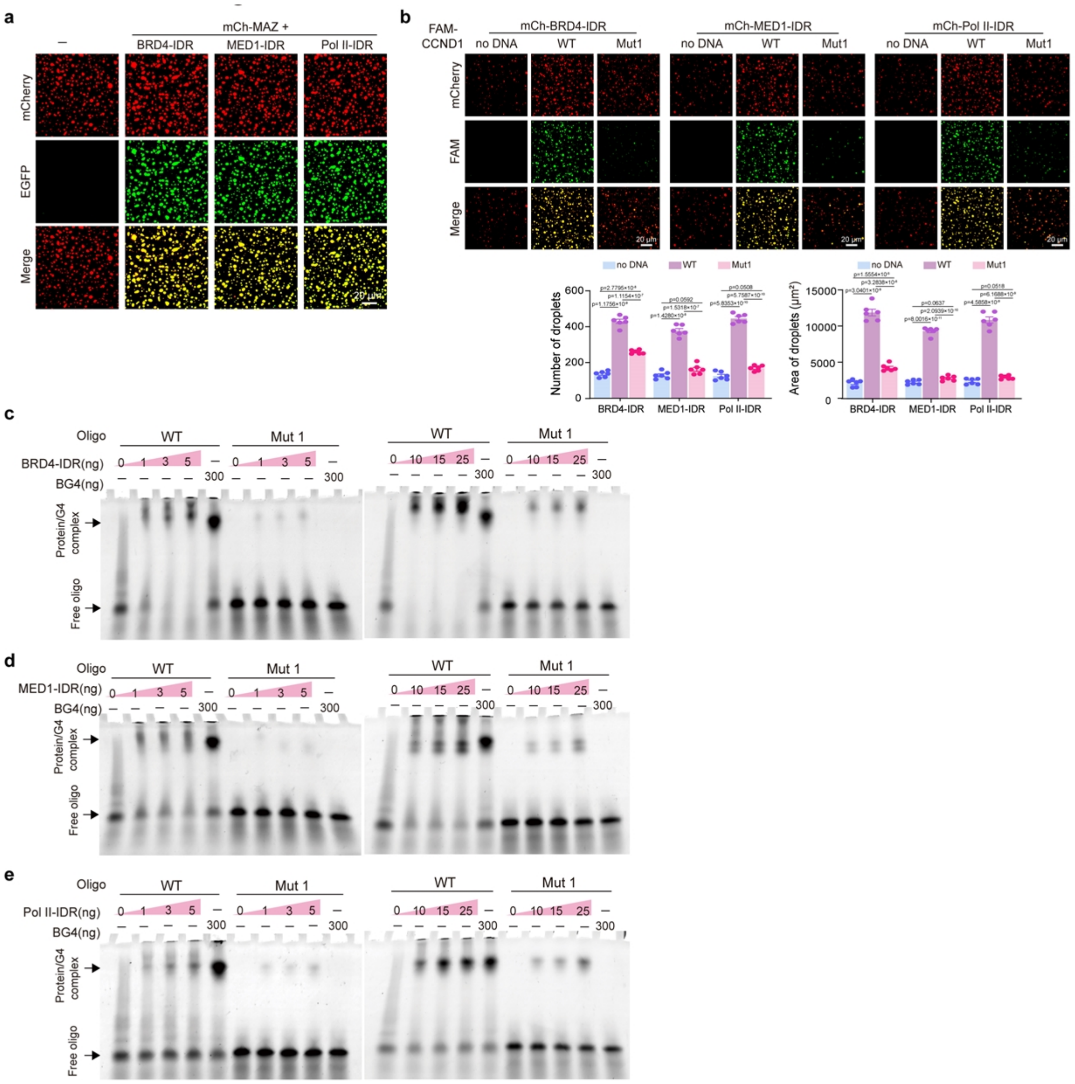

Supplementary Figure 15. Analyses of the IDRs of BRD4, MED1 and Pol II for their droplet formation and EMSA in the absence and presence of CCND1-G4 WT or Mut1 oligo.

a Incorporative droplet formation by mCherry-MAZ and EGFP-BRD4-IDR, EGFP-MED1-IDR, or EGFP-Pol II-IDR protein. Scale bar, 20 μm. Representative of three biologically independent experiments.

b Effects of FAM-labeled CCND1-G4 WT and Mut1 oligos on the droplet formation of EGFP-BRD4-IDR, EGFP-MED1-IDR, or EGFP-Pol II-IDR protein *in vitro*. Quantification of droplets' numbers and area are shown at right. Data are mean ± s.e.m. (n = 6 fields each). Unpaired two-tailed Student's t-test was used for statistical analysis. Scale bar, 20 μm.

c-e Increasing amounts of purified recombinant IDRs of BRD4 (**c**), MED1 (**d**) and Pol II (**e**) were individually incubated with FAM-labelled WT or Mut1 annealed in KCl-containing solution. BG4 was used as a positive control. Representative of three biologically independent experiments.

Source data are provided as a Source Data file.

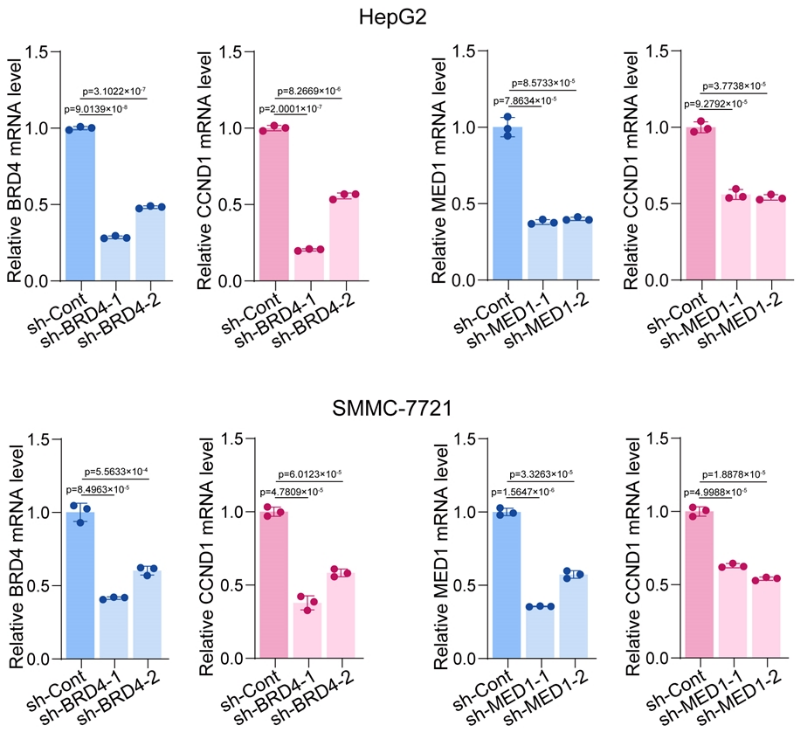

Supplementary Figure 16. Reverse transcription quantitative PCR (RT-qPCR) analysis of gene expression knockdown.

HepG2 (top panel) and SMMC-7721 (bottom panel) cells were individually infected by lentiviruses carrying shRNAs against a control sequence (sh-Cont), BRD4 (sh-BRD4-1 and sh-BRD4-2), or MED1 (sh-MED1-1 and sh-MED1-2) for 72 h. Total RNAs were extracted from the infected cells followed by RT-qPCR to determine the mRNA levels of BRD4, MED1 and CCND1 using their corresponding primers (listed in Supplementary Table 3). Data are mean ± s.d. (n = 3 biologically independent experiments). Unpaired two-tailed Student's t-test was used for statistical analysis. Source data are provided as a Source Data file.

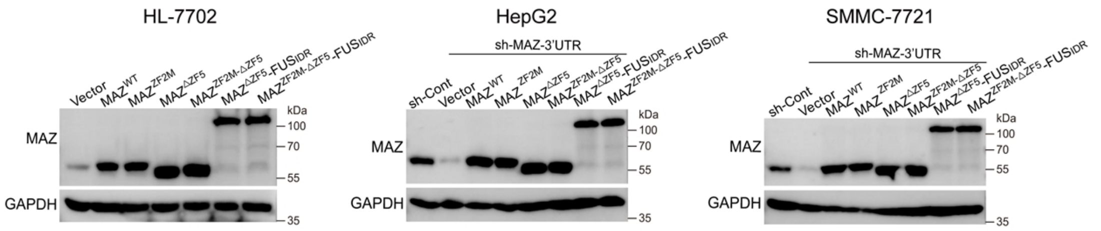

Supplementary Figure 17. Western blot analysis of MAZ WT and mutant proteins.

HL-7702 (left), HepG2 (middle) and SMMC-7721 (right) cells were individually infected by lentiviruses expressing MAZ WT and its mutants, either alone (in HL-7702 cells) or together with lentiviruses carrying shRNA targeting MAZ mRNA 3'-UTR (i.e., sh-MAZ-3'UTR), as indicated on the top of images. Expression of the exogenous MAZ and endogenous MAZ were tested by Western blot using the MAZ antibody with GAPDH as a loading control. Representative of three biologically independent experiments.

Source data are provided as a Source Data file.

Supplementary Tables

Supplementary Table 1. The information of antibodies used in the study.

Antibodies	Sources	Identifiers	Dilution rates
BG4	Absolute Antibody	cat# Ab00174-1.1	IF (1:500), ChIP (2 μg)
MAZ (133.7)	Santa Cruz	cat# sc-130915	WB (1:2000), IF (1:500), ChIP (2 μg)
MAZ	Proteintech	cat# 21068-1-AP	IF (1:500)
GAPDH	Acton	cat# 10R-G109A	WB (1:1000)
BRD4	Santa Cruz	cat# sc-518021	WB (1:200), IF (1:100)
MED1	Santa Cruz	cat# sc-74475	WB (1:200), IF (1:100)
CDK9	Cell Signaling Technology	cat# 2316	WB (1:1000), IF (1:500)
RNA Pol II S2P	Millipore	cat# 04-1571	WB (1:500), IF (1:200)
RNA Pol II S5P	Millipore	cat# 04-1572	WB (1:500), IF (1:200)
H3K4me3	Cell Signaling Technology	cat# 9751	WB (1:2000), IF (1:500)
H3K27ac	Abcam	cat# ab4729	WB (1:1000), IF (1:500)
H3K9me3	Cell Signaling Technology	cat# 13969	WB (1:1000), IF (1:500)
Lamin B1	Affinity	cat# AF5161	WB (1:1000)
Ki-67	Thermo Fisher Scientific	cat# 710229	IF (1:500)
Goat anti-Rabbit IgG (H+L) Highly Cross-Adsorbed Secondary Antibody, Alexa Fluor 488	Thermo Fisher Scientific	cat# A32731	IF (1:500)
Goat anti-Rabbit IgG (H L) Highly Cross-Adsorbed Secondary Antibody, Alexa Fluor 594	Thermo Fisher Scientific	cat# A32740	IF (1:500)
Goat anti-Mouse IgG (H L) Highly Cross-Adsorbed Secondary Antibody, Alexa Fluor 488	Thermo Fisher Scientific	cat# A32723	IF (1:500)
Goat anti-Mouse IgG (H+L) Highly Cross-Adsorbed Secondary Antibody, Alexa Fluor 594	Thermo Fisher Scientific	cat# A32742	IF (1:500)
Goat anti-Rabbit IgG (H+L) Secondary Antibody, HRP	Thermo Fisher Scientific	cat# A31460	WB (1:5000)
Goat anti-Mouse IgG (H+L) Secondary Antibody, HRP	Thermo Fisher Scientific	cat# A31430	WB (1:5000)

Supplementary Table 2. Sequences of FAM-labeled DNA probes, and wide type or mutant competitors.

Predicted MAZ binding sites and their mutants	Names of probes or competitors	Sequences (5' to 3')
BS1	BS1-For	AGGGGGAGGGGGCG
	BS1-Rev	CGCCCCCTCCCCCT
BS2	BS2-For	AGCGGGAGGGGGGC
	BS2-Rev	GCCCCCCTCCCGCT
BS3	BS3-For	GGGGGGCGGGGGCG
	BS3-Rev	CGCCCCCGCCCCCC
BS4	BS4-For	AATGGGAGCGGGAG
	BS4-Rev	CTCCCGCTCCCATT
BS5	BS5-For	GGGCGCAGGGGGAG
	BS5-Rev	CTCCCCCTGCGCCC
BS6	BS6-For	AGGGGGCGCGGGCG
	BS7-Rev	CGCCCGCGCCCCCT
BS1M	BS1M-For	AGGGTGAGTGGGCG
	BS1M-Rev	CGCCCACTCACCCT
BS2M	BS2M-For	AGCGTGAGTGGGGC
	BS2M-Rev	GCCCCACTCACGCT
BS3M	BS3M-For	GGGGTGCGTGGGCG
	BS3M-Rev	CGCCCACGCACCCC
BS123M	BS123M-For	ATGGGAGCGGGAGTGGTGCGTGGGCGGGCGCAG GGTGAGTGGGCGCGGGCG
	BS123M-Rev	CGCCCGCGCCCACTCACCCTGCGCCCGCCCACGC ACCACTCCCGCTCCCAT
ds-CCND1 or FAM-CCND1	FAM-ds-CCND1-For	ATGGGAGCGGGAGGGGGGCGGGGGCGGGCGCAG GGGGAGGGGGCGCGGGCG
	ds-CCND1-Rev	CGCCCGCGCCCCCTCCCCCTGCGCCCGCCCCCGC CCCCCTCCCGCTCCCAT
ds-CCND1-M	FAM-ds-CCND1-M-For	ATGAGAGCGAGAGAGAGACGAGAGCGAGCGCAG AGAGAGAGAGCGCGAGCG
	ds-CCND1-M-Rev	CGCTCGCGCTCTCTCTCTCTGCGCTCGCTCTCGTC TCTCTCTCGCTCTCAT
	FAM-Poly A	AAAAAAAAAAAAAAAAAAAAAAAAAA
	FAM-MYC-G4	TGAGGGTGGGTAGGGTGGGTAA
	FAM-TEL-G4	TAGGGTTAGGGTTAGGGTTAGGGTTAG
	FAM-BCL2-G4	FAM-GGGCGCGGGAGGAATTGGGCGGG
	FAM-MDM2-G4	GGGGCGCGGGGCGCGGGGCATGGGG
	FAM-MYB-G4	GGAGGAGGAGGAGGAGGAGGA

Supplementary Table 3. Primers used in RT-qPCR and ChIP assays.

Name	Sequence (5' to 3')	Size (bps)	Purpose
MAZ-For	GTTTCCTTGCACGCTGCTG	150	RT-qPCR
MAZ-Rev	GCAAAGAAGCGGGACTGGAG		
CCND1-For	GAAGGAGACCATCCCCCTGA	146	RT-qPCR
CCND1-Rev	CAATGAAATCGTGCGGGGTC		
BRD4-For	GTCCTATGAGGAGAAGCGGC	155	RT-qPCR
BRD4-Rev	GACGGCTTCAGGGTCTCAAA		
MED1-For	GGGGAGAATCCTGTGAGCTG	271	RT-qPCR
MED1-Rev	AAATGACCCCCACTCCTTGG		
GAPDH-For	GCCTTCCGTGTTCCTACC	96	RT-qPCR
GAPDH-Rev	CTTCACCACCTTCTTGATGTC		
CCND1-Prmt-For	AAAACCGGACTACAGGGGCAACTC	167	ChIP
CCND1-Prmt-Rev	AAAGATCAAAGCCCGGCAGAGAAT		

Supplementary Table 4. Sequences of forming G4s with different numbers of G-quartets and loop lengths.

Name	Sequence (5’ to 3’)
G2T1	FAM-GGTGGTGGTGG
G2T2	FAM-GGTTGGTTGGTTGG
G2T3	FAM-GGTTTGGTTTGGTTTGG
G2T4	FAM-GGTTTTGGTTTTGGTTTTGG
G2T5	FAM-GGTTTTTGGTTTTTGGTTTTTGG
G3T1	FAM-GGGTGGGTGGGTGGG
G3T2	FAM-GGGTTGGGTTGGGTTGGG
G3T3	FAM-GGGTTTGGGTTTGGGTTTGGG
G3T4	FAM-GGGTTTTGGGTTTTGGGTTTTGGG
G3T5	FAM-GGGTTTTTGGGTTTTTGGGTTTTTGGG
