# Supplementary material for: G-quadruplexes promote the motility in MAZ phase-separated condensates to activate CCND1 expression and contribute to hepatocarcinogenesis
Source: Nat Commun. 2024 Feb 5;15:1045. doi: 10.1038/s41467-024-45353-5 (PMC10844655; doi:10.1038/s41467-024-45353-5)
Supplement: Supplementary file 3 — Description of Additional Supplementary Files [file 41467_2024_45353_MOESM3_ESM.pdf]

Description of Additional Supplementary Files

File Name: Supplementary Movie 1
Description: A movie showing fusion process of mCherry-MAZ droplets *in vitro.*

File Name: Supplementary Movie 2
Description: A movie showing fusion process of mCherry-MAZ puncta in cell nucleus.

File Name: Supplementary Movie 3
Description: A movie showing fusion process of mCherry-MAZ^{ZF3-5} droplets *in vitro.*

File Name: Supplementary Movie 4
Description: A movie showing fusion process of MAZ/CCND1-G4 droplets formed by mCherry-MAZ and CCND1-G4 structures *in vitro.*
